# Personal

**Published:** 1909-03

**Authors:** 


### PERSONAL.

DR. JOSEPH M. MATHEWS, of Louisville, has resigned the presidency of the Kentucky State Board of Health, a post he held for seventeen years. Resolutions of regret were adopted by the Jefferson County Medical Society, coupled with a request that Dr. Mathews be requested to reconsider his action. Dr. and Mrs. Mathews are in Southern California for the winter.

---

Dr. George G. Wagner, 86 Greenfield Street, Buffalo, has been appointed district physician in District No. 8, vice Dr. John A. Hoffmeyer resigned. Dr. Wagner formerly resided at Angola.

---

Dr. Patrick J. Hurley, of No. 2272 Seneca Street, Buffalo, was recently appointed a surgeon on the Mercy hospital staff. He has been until lately one of the internes in the surgical wards of Saint Johns' hospital, New York.

---

Dr. Eugene H. Porter, of New York, has been reappointed State Commissioner of Health. Dr. Porter was first appointed by Governor Higgins, and made a good record in his first term of service.

---

Dr. Jacob Goldberg, of Buffalo, was chosen unanimously to be Chairman of the Board of School Examiners at the meeting of the board held early in February. Dr. Goldberg has been a member of the board for some time past, and his selection as chairman is a gratifying recognition of his services.

---

Dr. Alexander Smith, of Rochester, has been appointed assistant physician at the Soldiers' and Sailors' Home, Bath, N. Y., vice Dr. Carlton Foster resigned.

---

Dr. Edward L. Frost, Buffalo, has been appointed specialist in children's diseases on the staff of the Riverside Hospital.

---

Dr. William R. Davidson, of Evansville, Ind., has become associated in practice with Dr. Edwin Walker, and in the joint management of Dr. Walker's sanitarium in that city.

---

Dr. E. A. Bowerman, of 234 E. Utica Street, Buffalo, has been appointed Police Surgeon, vice Dr. Joseph Fowler, deceased. This appointment reflects credit upon the Board of Police Commissioners, it being regarded on all hands as an exceptionally good one, Dr. Bowerman being well qualified for the post.

